# The impact of tidal regimes on the stress physiology of English oak (*Quercus robur)*

**DOI:** 10.3389/fpls.2026.1810933

**Published:** 2026-06-10

**Authors:** Katharina Wilfert, Stijn Baeten, Els Prinsen, Tom Maris, Jonas Schoelynck

**Affiliations:** 1 Ecosphere Research Group, Department of Biology, University of Antwerp, Antwerp, Belgium; 2 Integrated Molecular Plant Physiology Research Group, Department of Biology, University of Antwerp, Antwerp, Belgium

**Keywords:** controlled reduced tide areas, estuarine restoration, hydrogen peroxide, mesocosm, stress physiology

## Abstract

**Introduction:**

Restored estuarine floodplains with an artificially controlled reduced tide (CRT) are dynamic areas created for flood protection whilst also providing habitats for estuarine wildlife. However, the reintroduction of the tide poses stressors, including salinity and waterlogged soils because of periodic flooding, on established vegetation such as English oak (*Quercus robur*). Therefore, newly opened CRT areas give the opportunity of studying the effects of flooding, salinity, and drainage, on hydrogen peroxide (H_2_O_2_) concentrations in plants, as a proxy for stress.

**Methods:**

This was done on English oak samples retrieved from both field and mesocosm settings using commercially available peroxide assay kits.

**Results:**

Our results indicated that H_2_O_2_ concentrations measured in the field were affected by the time of sampling, while concentrations in the mesocosm were influenced by soil type. Flooding and salinity did not show detectable effects on H₂O₂ concentrations, either individually or in combination with soil type, within the scope of our experimental design, which had constrained statistical power to detect subtle effects. Additionally, our results did not correspond with the deterioration and mortality of English oak observed in the field and mesocosm experiments, indicating limited suitability of H₂O₂ as a standalone indicator of physiological stress under these conditions.

**Discussion:**

Overall, we recommend that future studies include a broader range of indicators to assess the tree stress responses in dynamic CRT environments.

## 1 Introduction

Terrestrial plants are frequently exposed to abiotic stressors of both natural and human nature. Their limited mobility restricts options to combat these stressors, so they must change their morphology and/or physiology accordingly or risk perishing under the increased stress. In an ideal scenario, plants have ample time to adapt to the new conditions, but this is not always the case. In complex dynamic ecosystems, many stressors are present at the same time often leading to antagonistic or synergistic effects, permanently altering the physiology and survival of the exposed individuals (Renziehausen et al., 2024).

An example of environments with sudden high stressor dynamics are restored estuarine floodplains with an artificially controlled reduced tide (CRT). These CRT areas are reclaimed land given back to nature serving two purposes: flood control and estuarine wildlife habitat creation (Maris et al., 2007). However, the transition from former land use to CRT is usually not advantageous for most of the species already present. Flooding, increased salinity and waterlogged soils are new stressors imposed on the former terrestrial vegetation, such as the English oak (*Quercus robur L.*), a widespread and ecologically as well as economically valuable tree species in Belgium. Many of these oaks were already established prior to the restoration and can be several decades old. This raises the question whether English oak will perish under the new and dynamic conditions of estuarine CRT areas and what the driving factors are.

In North America and Europe different oak species are known to grow in freshwater wetlands and floodplains. Previous studies have identified that the type of flooding and flood duration are crucial factors for oak tree survival (Kabrick et al., 2012; Angelov et al., 1996; Anderson and Pezeshki, 2001), impacting many metabolic mechanisms (e.g. gas exchange, photosynthesis, growth rate) as well as morphological traits (e.g. leaf number, leaf area) (Anderson and Pezeshki, 1999; Dreyer et al., 1991; Anderson and Pezeshki, 2001). In CRT areas intermittent flooding is the most common type and is believed to have a lower impact on tree survival compared to continuous flooding (Angelov et al., 1996). Additionally, impacts of flooding can be exacerbated by decreased soil drainage (e.g. soil composition, soil compaction, etc.), making it an important aspect of CRT areas.

Depending on the CRT location in the estuary, salinity of the flooding water is another stressor directly resulting from transitions of euhaline water downstream, over polyhaline, mesohaline and oligohaline mixing zones, towards freshwater in the most upstream parts of the estuary (Meire et al., 2005). Much like flooding, salinity affects metabolic mechanisms (Gugliuzza et al., 2023; Sehmer et al., 1995; Singh and Stasolla, 2016), possibly exacerbating the impact of flooding. Singh and Stasolla (2016) for example, observed a positive correlation between salinity and leaf damage of *Quercus macrocarpa* Michx. and *Quercus rubra* L., by measuring the reactive oxygen species hydrogen peroxide. Conner et al. (1998) noticed that flooding and salinity combined reduce growth, biomass and photosynthesis in four different oak tree species, thereby altering physiology and survival chance.

Over the past decade hydrogen peroxide (H_2_O_2_) has increasingly been proposed as proxy for abiotic stress measurements. It is known as stress signaling molecule in several signal transduction pathways of terrestrial plants (Taiz et al., 2018; Apel and Hirt, 2004) including oaks, and can accumulate in plant tissues over time (Pasternak et al., 2005; Kankanamge et al., 2011) if scavenger mechanisms are exhausted. However, accumulation may differ between the types of tissue sampled, the sampling time and the type of stress applied, raising the question whether H_2_O_2_ can reliably reflect the stress induced in oak trees found in CRT areas.

Several studies have used H_2_O_2_ in the past to evaluate stressors with a temporal component, such as heat stress (Sharma et al., 2012), excessive solar radiation (Asaeda et al., 2023), flooding (Asaeda et al., 2022), and salinity exposure (Sehmer et al., 1995). Given the practical constraints of CRT areas with respect to the limited time window for access and sampling methodologies, H_2_O_2_ could be a convenient parameter compared to more established indicators (e.g. phytohormones, gas exchange, water potential, etc.), especially regarding the amount of sample, the aperture and laboratory analysis required.

The present study will therefore investigate two aspects. First, it will evaluate the effect of flooding, as well as the combined effects of flooding, salinity, and drainage, on hydrogen peroxide (H_2_O_2_) concentrations in English oak, to assess their adaptivity to the dynamic conditions of CRT areas. Secondly, this study will explore if H_2_O_2_ can capture the suspected effects and be used as a reliable proxy for broader stress responses in the future. The findings are expected to improve our understanding of stress patterns observed in field sites such as CRT areas and to clarify how environmental changes in these systems affect established terrestrial vegetation.

## 2 Methodology

### 2.1 Field campaign

In 2017, the presence of living English oak (*Quercus robur*) trees within three areas under CRT were mapped (Bergenmeersen, Kruibeke, and Zennegat), and these sites, expanded with a fourth, newly created CRT (Grote Vijver), were revisited during the summer of 2022 (**Table 1**). During the revisit, we recorded which trees were still alive and which had died. Based on the locations with both living and dead trees, different tree locations were selected using a stratified random sampling approach for more intensive sampling. This intensive sampling campaign took place in autumn 2022 and autumn 2023.

**Table 1:** Summary of key characteristics of the four studied controlled reduced tide areas.

CRT area	Under tidal influence since	Co-ordinates	Flooding frequency (%)	Range of water table during flooding (m)	Flood duration (min)	Soil conductivity 5-10cm below ground (µS/cm)	Diameter (cm) as proxy for tree age
Bergenmeersen	2014	51° 1’4.08”N 3°58’11.21”E	2 - 74	0.15 – 0.20	122 – 155	321 – 1781	0.30 – 1.90
Grote Vijver	2021	51° 4’14.62”N 4°25’39.46”E	7 - 96	0.21 – 0.74	129 – 313	837 – 3483	0.30 – 14.60
Kruibeke	2017	51° 9’56.87”N 4°19’12.13”E	0 - 98	0.00 – 0.37	0 – 832	610 – 6380	3.20 – 45.20
Zennegat	2017	51° 3’20.42”N 4°26’18.17”E	34 - 39	0.11 – 0.12	130 – 134	1424 – 1742	0.30 – 1.60

Data for flood frequency, range of water table during flooding and flood duration were extracted from Waterinfo (2023) as described below. Soil conductivity and diameter at breast height were measured in the field.

During two sampling campaigns, one in September 2022 and another in September 2023, it was again recorded whether trees were alive or dead. Then five mature, turgid leaves were randomly sampled from each oak tree (n=49) and immediately stored in a cryoshipper filled with liquid nitrogen. Leaf samples were brought to the lab and stored at -80 °C awaiting further analysis. In addition to leaf sampling, total-leaf area (TLA) (Jacobs et al., 2013) and Londo-scale estimates were taken for every tree. Trees characterized by a lower TLA or Londo score were presumed to be pre-stressed. Furthermore, tree height was estimated for all trees. Additionally, the diameter at breast height of each tree was noted as proxy for tree age, which varied between CRT areas. The ground surface elevation was measured at each tree location using an RTK-GPS (Trimble R8s GNSS receiver with TSC3 controller) and recorded in meters relative to TAW (Tweede Algemene Waterpassing) which is the Belgian reference height used for topographic and bathymetric elevation measurements. A height of 0 m TAW corresponds to the mean low water level at the city of Ostend. Additionally, the distance to the nearest creek was measured, which is the main route bringing or draining water during flood and ebb phases, respectively. Finally, six-water-related and 18 soil-related parameters were evaluated in each CRT area (**Table 2**) according to the methodologies below.

**Table 2:** List of tree-, water- and soil-related parameters measured in the field or extracted from Waterinfo (2023).

Type of parameter	List of parameters			
			
Water-related parameters	Inundation time (min)	Inundation height (m)	Inundation frequency (%)
	Elevation (m)	AV dry time (min)	Max dry time (min)
Soil-related parameters	Clay content 5cm below ground (%)	Clay content 25cm below ground (%)	Silt content 5cm below ground (%)
	Silt content 25cm below ground (%)	Sand content 5cm below ground (%)	Sand content 25cm below ground (%)
	Organic matter content 5cm below ground (%)	Organic matter content 25cm below ground (%)	Dry bulk density 5cm below ground (g/cm³)
	Dry bulk density 25cm below ground (g/cm³)	Water content 5cm below ground (%)	Water content 25cm below ground (%)
	Gravimetric water content 5cm below ground (g/g)	Gravimetric water content 25cm below ground (g/g)	Volumetric water content 5cm below ground (%)
	Volumetric water content 25cm below ground (%)	Conductivity in the soil 5-10cm below ground (µS/cm)	Conductivity in the soil 25-30cm below ground (µS/cm)

For each tree location (n=49), six undisturbed soil cores were collected using Kopecky rings (stainless steel rings with a fixed volume of 100cm³), three at depths 5 to 10 cm and three at depths 25 to 30 cm below the surface. Subsequently, a subsample was taken from each depth for granulometric analysis. Water levels within the CRT’s were downloaded from Waterinfo (Waterinfo, 2023). For each individual location, the Inundation time (min), Inundation height (m), Inundation frequency (%), Average dry time (min), and Maximum dry time (min) were calculated using the tidal data and the Tides R package (Cox and Schepers, 2018) in R version 4.2.2 (R Core Team, 2022). These parameters were computed based on the data (2022 or 2023) ranging from the sampling day up to one-year prior sampling to capture the total variation in tidal activity throughout the seasons.

The six undisturbed soil samples per tree location were weighed, subsequently oven-dried at 70 °C for one week, and weighed again. By subtracting the dry weight from the wet weight, the Volumetric water content (%) and the Gravimetric water content (g/g) were calculated for each sample. Furthermore, the dry weight was used to calculate the Bulk dry density (g/cm³). The dried sample was then homogenized and sieved over a one-millimeter mesh, after which the sample was used to determine the specific Electrical conductivity of the soil (µS/cm) and the Organic matter content (%). To measure the electrical conductivity of the soil, 10g of dry matter was mixed with 25ml of de-ionized water and shaken for 30 minutes, followed by a 15-minute settling period. After settling, the electrical conductivity of the supernatant was determined using a WTW Multi 3430 equipped with a Tetracon 925 probe. Organic matter content was determined using the Loss on Ignition (LOI) method (Heiri et al., 2001) at 550 °C for five hours. Finally, an average of each result was calculated per depth and location.

Subsamples for grain size analysis were pretreated by heating after the addition of H_2_O_2_ and HCl to remove organic matter and dispersed using ultrasound. The volume-weighted mean grain size and the percentages of clay (<2µm), silt (2-63µm), and sand (>63µm) (Gordon et al., 1992) were determined using the laser diffraction method with a Malvern Mastersizer 2000.

### 2.2 Mesocosm experiment

In spring of 2023 ninety English oak (*Quercus robur*) trees were ordered from a commercial plant nursery and planted in soil excavated during the construction of a depoldering site along the freshwater part of the Scheldt River. Twenty-three trees were grown in a homogenized silty loam soil, while 67 trees were grown in a mixture of 60% homogenized silty loam with 40% of gravel (>2mm). The excavated soil was characterized by a dry bulk density of 1.36 g/cm^3^ (silty loam only), and a median grainsize of 26μm, consisting of 5% clay, 70% silt, and 25% sand. The nutrient content of the soil was: 4.80mg N/kg for NO_3_-N, 7.50mg N/kg for NH_4_-N, 1244.40mg/kg for total N, 38.60mg P/kg for PO_4_-P, 737.60mg/kg for total P, and 7177.60mg/kg for K. Trees were left to acclimate for one year and watered occasionally. After one year, tree circumference was measured 10cm above the soil and ranged from 2.40 to 5.60cm. We will refer to these trees as young oak trees from here on out.

The following spring (May 2024) pots were moved to mesocosms and distributed ad random into nine boxes with an electrical conductivity of either 500 or 3000µS/cm, which will be referred to as low or high salinity treatment from here on out. These concentrations were achieved using a mix of predominantly NaCl and a small amount of NaHCO_3_ to stabilize pH.

Three of the nine boxes had no inundation frequency (NIF) regime and were watered regularly. Inundation for the other six boxes was created using two large water tanks pumping water into the experimental boxes where it remained for six hours before being pumped out again. This cycle was repeated twice per day comparable to a natural tidal regime. Trees in the six boxes were placed onto stainless steel inserts either 25 or 50 cm above the box floor. Pots at 25 cm were defined as high inundation frequency (HIF) group, while the pots at 50 cm were defined as the low inundation frequency (LIF) group. The HIF group was flooded twice daily (flooding frequency 100% for 360 consecutive minutes) maintaining a water level approximately 10 cm above the soil surface. The LIF group only flooded twice during two days of the week (flooding frequency approx. 30% for 360 consecutive minutes) also maintaining a water level approximately 10 cm above the soil surface. During these so-called spring tides, the water level at the HIF site increased to 35 cm above the soil surface.

**Figure 1:**
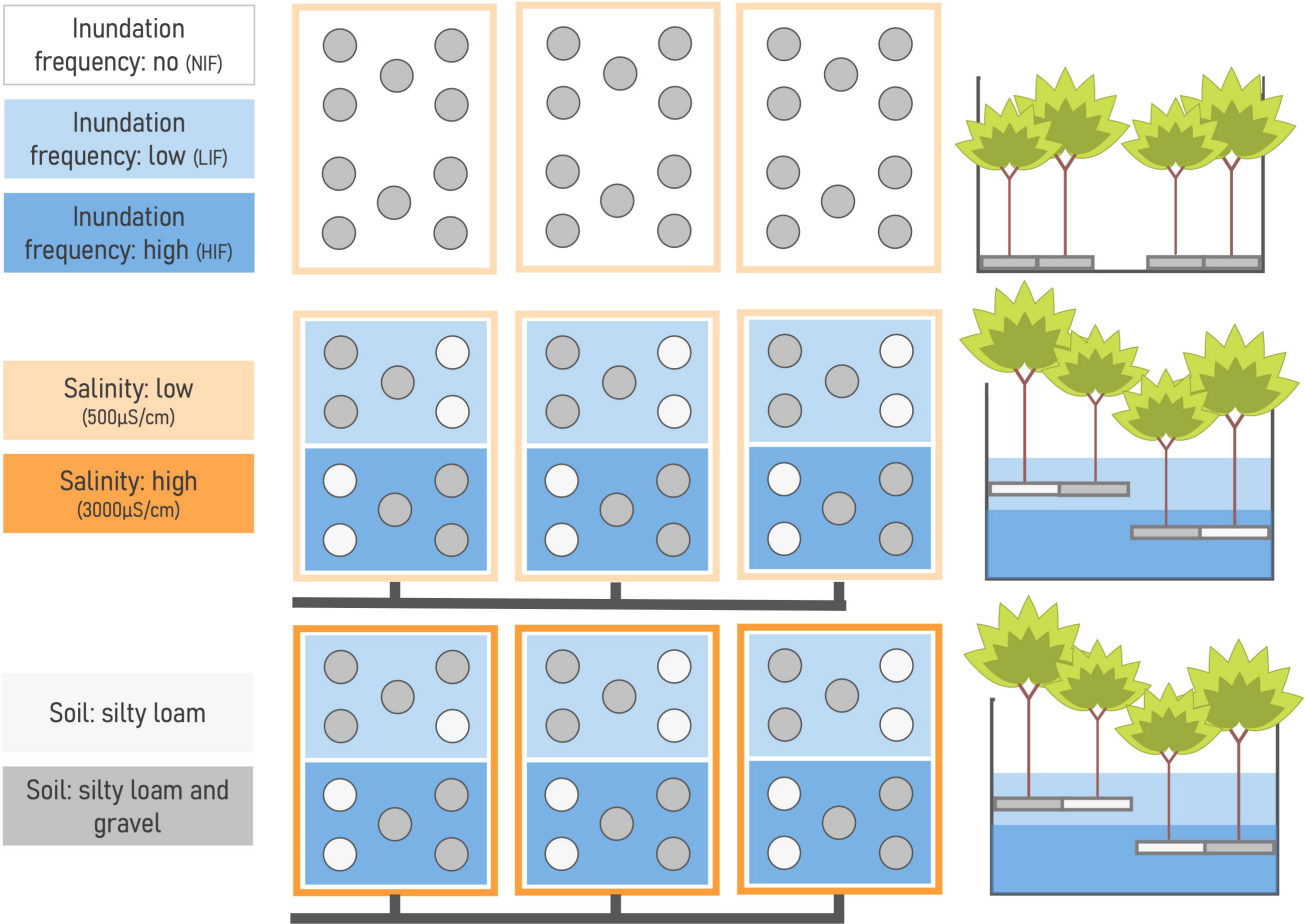
Schematic overview of the experimental setup including inundation regimes, salinity groups and soil groups. The nine boxes are shown from a top view with each circle representing one tree. On the right-hand side a side view of the boxes is provided, to show water levels during the flooding regimes.

Additionally, to the inundation regimes, trees in the six boxes were split into two soil groups: silty loam and silty loam mixed with gravel. This led to nine different treatment groups, which were investigated in this study (**Figure 1**): NIF-500µS/cm-silty loam and gravel (n=30), LIF-500µS/cm-silty loam (n=6), LIF-500µS/cm-silty loam and gravel (n=9), HIF-500µS/cm-silty loam (n=6), HIF-500µS/cm-silty loam and gravel (n=9), LIF-3000µS/cm-silty loam (n=5), LIF-3000µS/cm-silty loam and gravel (n=10), HIF-3000µS/cm-silty loam (n=6), and HIF-3000µS/cm-silty loam and gravel (n=9). These factor levels were chosen based on the field sites, to capture as much of the natural variation as possible.

Once emerging leaves had developed enough at the start of June (2024), monthly photos of five healthy leaves were taken and their color was determined using hex color codes. While this approach is less robust than some alternatives, it provided a more objective measure than relying solely on expert judgement. In September three to five leaves (depending on the total amount of good leaves available) were removed from every tree, placed into liquid nitrogen, and stored in the freezer at -80°C awaiting further analysis. During the entire period temperature (°C), electric conductivity (µS/cm), pH (-), and oxygen (mg/L) content were monitored regularly.

### 2.3 Hydrogen peroxide analysis

Hydrogen peroxide concentrations in leaves from the field and mesocosm experiment were quantified using the Sigma-Aldrich Peroxide Assay Kit (MAK311) with a detection range of 0.2–30µM H_2_O_2_ in a 96-well format. To do so, leaves were removed from the freezer and ground in liquid nitrogen using a mortar and pestle. For each tree five leaves where ground and mixed to minimize variation in H_2_O_2_ concentrations stemming from between leave variation. Powdered material was then brought into suspension in MilliQ water, centrifuged (20 min, 15,000 g, 4 °C, in an Eppendorf 5810R centrifuge, Eppendorf, Hamburg, Germany), and detection reagent was added provided by the assay kit. A standard curve was added to the 96-well plate and absorbance was measured at 585nm (A585) (Synergy MX, Agilent Biotek, CA, US). Two technical replicates were measured per tree and the H_2_O_2_ concentration was calculated and expressed in nmol/gFW.

### 2.4 Statistics

#### 2.4.1 Field campaign

Prior to the Principal component analysis (PCA), all twenty-four predictors from the collected field data were assessed for multicollinearity. A pairwise Spearman correlation coefficients using the *chart.Correlation()* function from the *PerformanceAnalytics* package in R (with use = “complete.obs”) was computed. Highly correlated predictors (>0.75) were identified and removed. The remaining nine predictors (Clay content 5 and 25cm below ground, Conductivity in the soil 5-10cm below ground, Distance to the nearest creek, Dry bulk density 25cm below ground, Elevation, Inundation frequency, Organic matter content 5cm below ground, and Volumetric water content 25cm below ground) were additionally assessed for multicollinearity by calculating variance inflation factors (VIFs) from a linear model using the *vif* function from the *car* package in R. None of the predictors exceeded the VIF cut-off value of ten.

Multivariate outliers were assessed using Mahalanobis distances, computed from the correlation matrix and its covariance structure. Observations exceeding the 99% quantile of the χ² distribution were considered potential outliers, however, none were detected. All predictor variables were then standardized (mean-centered and scaled to unit variance) prior to the PCA analysis, as they differed substantially in magnitude, log transformation was not required. The PCA analysis was conducted on the standardized predictor variables using the *prcomp()* function including confidence ellipses (95%) to explore the main gradients of variation in the field data. PCA was used as an exploratory tool to assess variable contributions to dominant axes of variation. Based on variable loadings, a subset of ecological meaningful predictors representing the major gradients in the data was selected for inclusion in subsequent linear mixed-effects model, to evaluate the effect of the predictor parameters on H_2_O_2_ concentrations.

Prior to modelling, exploratory data screening was performed. Because raw outcome values were right-skewed a log-transformation was applied. The model included Year, Elevation, Inundation frequency, Bulk density, Clay content, and Conductivity in the soil, as fixed effects. One datapoint for which not all predictor values were available was removed (n=34). To account for repeated measurements, sampling point was added as a random effect. The model was fitted using the *lmer()* function from the *lme4* package in R. Restricted maximum likelihood (REML) was applied, model singularity was evaluated with the *isSingular()* function and residual diagnostics were conducted using the *DHARMa* package.

#### 2.4.2 Mesocosm experiment

Given the moderate sample size and the high site-to-site variance of the field data, we set up a mesocosm experiment to examine predictors under more controlled circumstances. We focused on inundation frequency (high, low or no), soil type (silty loam or silty loam and gravel) and salinity (500 or 3000 µS/cm), as these three stressors had the highest contribution to the PCA axes. Factor levels for the three stressors were chosen according to our field sites. Hydrogen peroxide was kept as outcome variable (2024) to allow for comparison between the field and experimental data.

Outliers were removed from the continues outcome variable using quartiles and the interquartile range before data was log-transformed to reduce the left skew. Two liner mixed-effects models were fitted using the *lmer()* function from the *lme4* package in R and restricted maximum likelihood (REML) was applied. For both linear mixed-effects models, box was added as random intercept to account for possible variation between them. The first model evaluated whether inundation frequency (high, low, or no) by itself affects H_2_O_2_ concentrations (n=56) while the second model investigated the impact of inundation frequency (low or high), salinity (500 or 3000µS/cm) and soil type (silty loam or silty loam mixed with gravel) on H_2_O_2_ concentrations (n=55). Assumption testing for all models was done using the *DHARMa* package.

Given the small sample size of the mesocosm setup and the multiple fixed effects measured a *post-hoc* (observed) power analysis was applied. Statistical power was estimated using a simulation-based approach with the R package *simr*. The fitted mixed-effects model was extended along the grouping factor *box* and power was calculated separately for each fixed effect (soil, level, and treatment) using likelihood ratio tests based on 1000 simulations per effect. Confidence intervals representing binomial uncertainty around the estimated power were noted down as well. As the simulations were based on the observed effect sizes from the fitted model, these estimates should be interpreted as *post hoc* (observed) power.

## 3 Results

### 3.1 Field campaign

**Figure 2:**
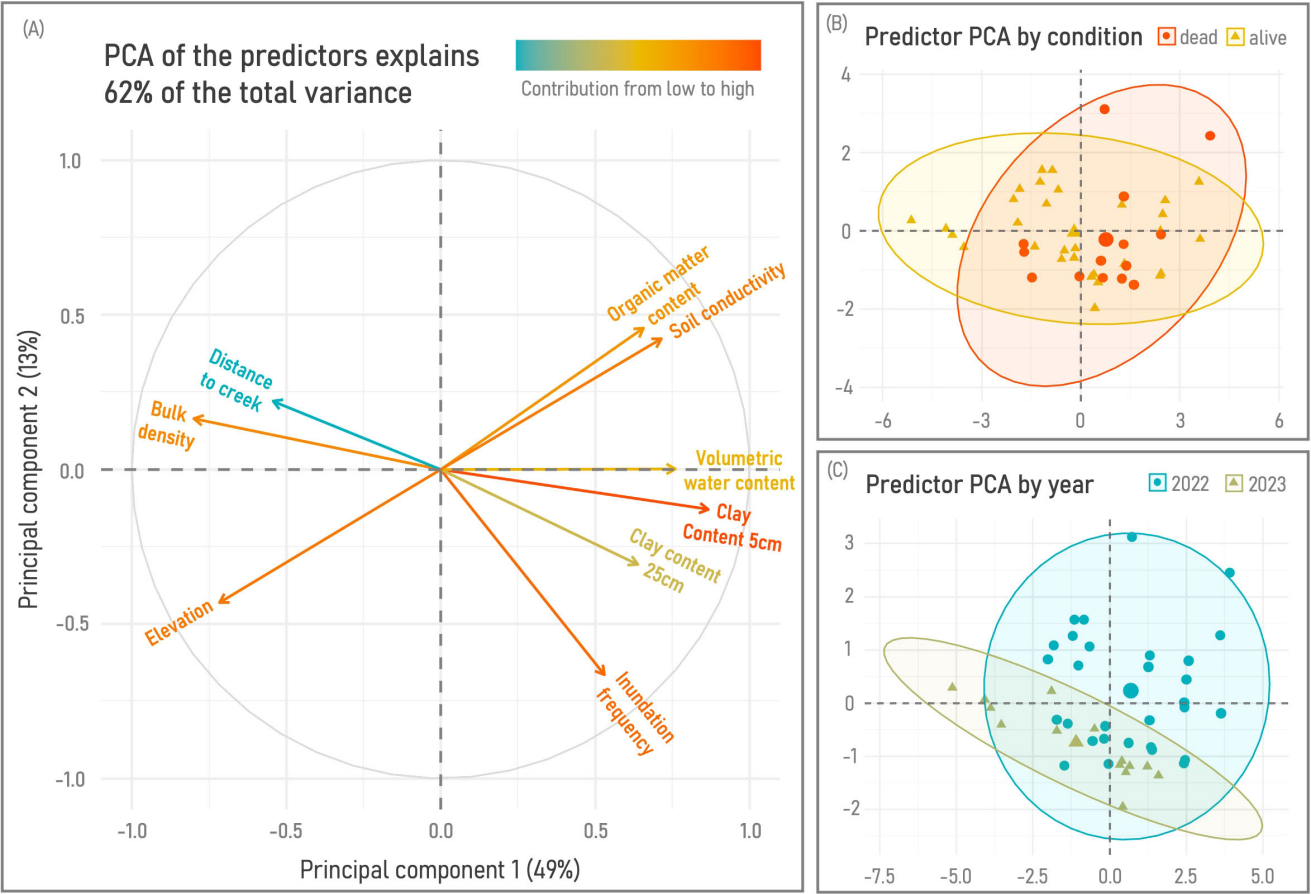
Panel **(A)** shows the Principal Component Analysis (n = 49) of the nine predictor variables. They are Elevation, Bulk density 25cm below ground, Distance to the nearest creek, Inundation time, Organic matter content 5cm below ground, Soil conductivity 5-10cm below ground, Volumetric water content 25cm below ground, Clay content 5 and 25cm below ground, and Inundation frequency. Panel **(B)** shows an overlay of condition onto the PCA indicating trees that are either dead (n=14) or alive (n=35). Panel **(C)** shows the overlay year of sampling.

From 2017 to 2023 we had increasingly visual cues that oak trees seemed to deteriorate in the CRT’s. While all 49 recorded trees were alive in 2017, only 35 of them were still alive by 2023, indicating that about thirty percent of the trees had died. Using field data from 2022–2023, the PCA analysis was used to explore the main gradients of variation in environmental predictors (**Figure 2**) and to inform the selection of variables for modelling H_2_O_2_ concentrations in oak leaves.

The first principal component (PC1) explained 49% of the total variance in the predictor set, while the second principal component (PC2) accounted for an additional 13% (**Figure 2A**). Together about 62% of the overall variance was captured. PC1 mainly distinguished areas with wetter soils from once with drier soils due to differences in elevation, distance to creek and multiple soil properties. PC2 was mainly influenced by inundation frequency. The PCA was then used to explore clustering with respect to tree condition (**Figure 2B**) and the year of sampling (**Figure 2C**). Some separation of living trees along PC1 was observed, suggesting a potential association with elevation and soil parameters (Panel B). The distribution of dead trees showed a broader spread and appeared more aligned with PC2, which may indicate a relationship with inundation frequency. However, these patterns should be interpreted cautiously as the PCA analysis was used as an exploratory tool only and no direct conclusions can be drawn from it. Strong clustering for samples collected in 2023 aligns with PC1 (Panel C). The ellipse for samples from 2022 on the other hand, is substantially larger indicating more variation within the samples. It also does not clearly align with one of the PCs.

**Figure 3:**
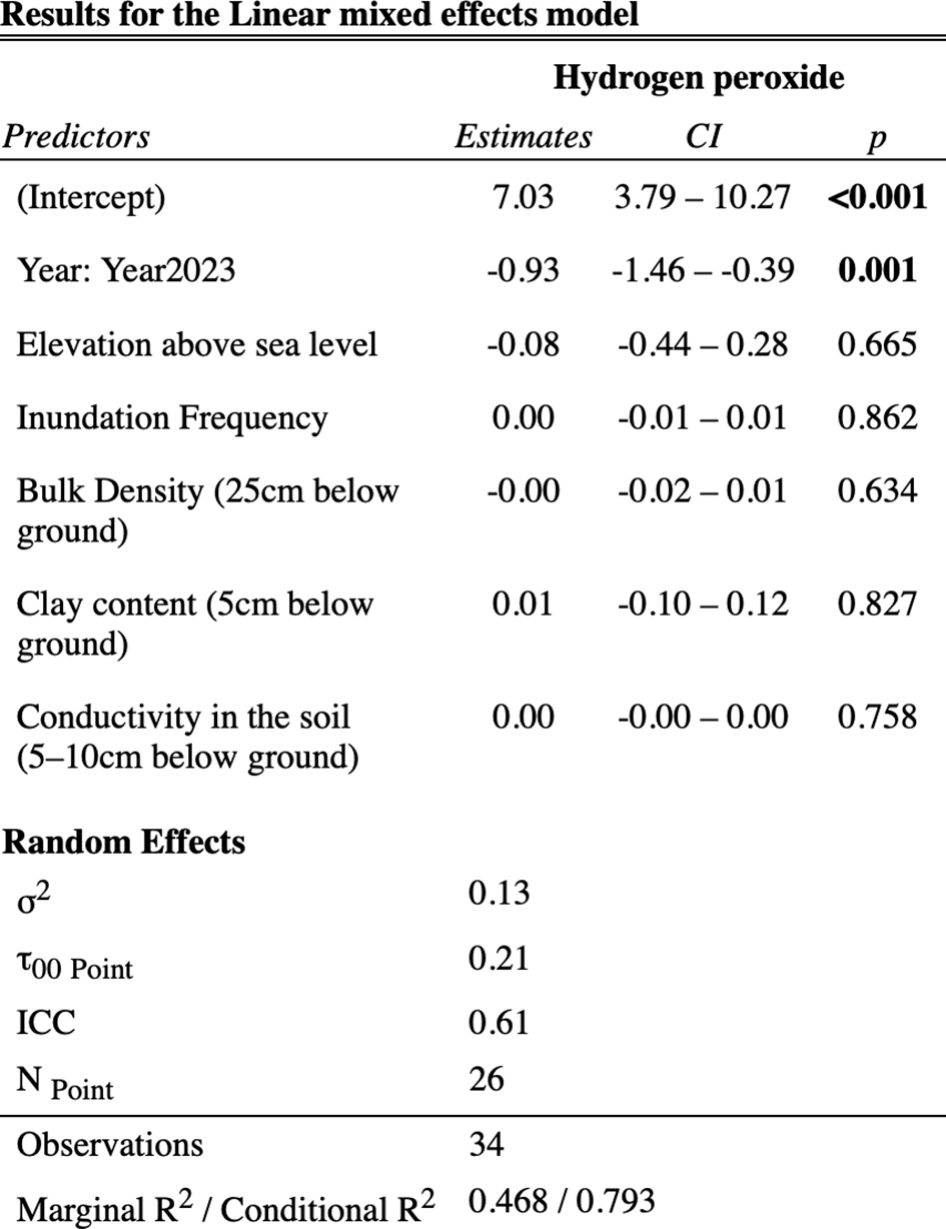
Results of the linear mixed-effects model (n=34) for the field data from 2022 and 2023.

The model intercept (representing estimated log-transformed H_2_O_2_ concentrations in 2022) was 7.03 (SE = 1.574, *t* = 4.468). From all the predictors (fixed effects) only Year had a significant effect on H_2_O_2_, with concentrations being significantly lower in 2023 than in 2022 (β = −0.926, SE = 0.258, *t* = −3.587, p = 0.001). The Intraclass Correlation Coefficient (ICC) of 0.61 indicates a large variance in H_2_O_2_ concentrations between Sampling points (random effect), suggesting strong site-to-site variation (**Figure 3**). Together, the limited number of significant predictors and the high unexplained variance indicate that important drivers of H_2_O_2_ accumulation were not fully captured by the predictors included in the model.

### 3.2 Mesocosm experiment

**Figure 4:**
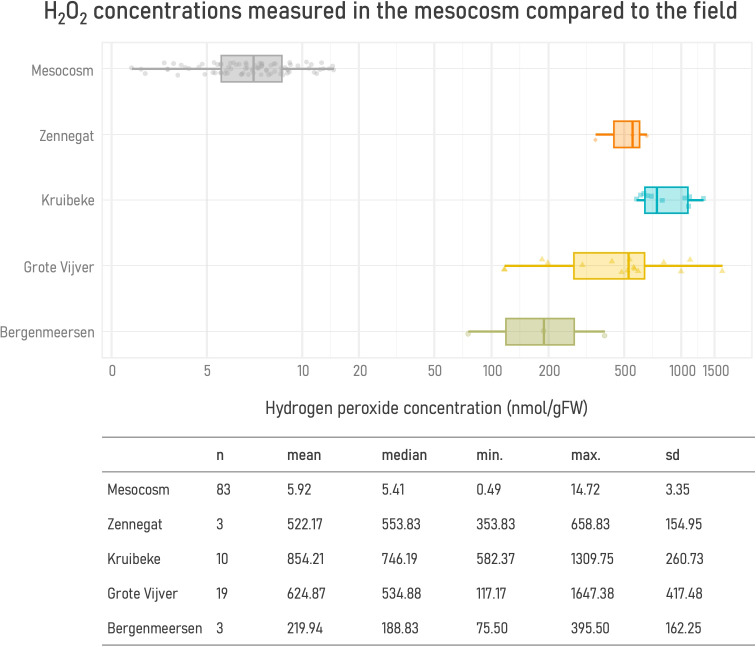
Hydrogen peroxide (H_2_O_2_) concentrations measured in oak trees from the mesocosm experiment (n=83) and from the different field sites (n=35). Data is plotted on a pseudo log-scale. The table shows the number of observations (n), the mean, the median, the lowest measurement and the highest measurement, as well as the standard deviation (sd).

**Figure 5:**
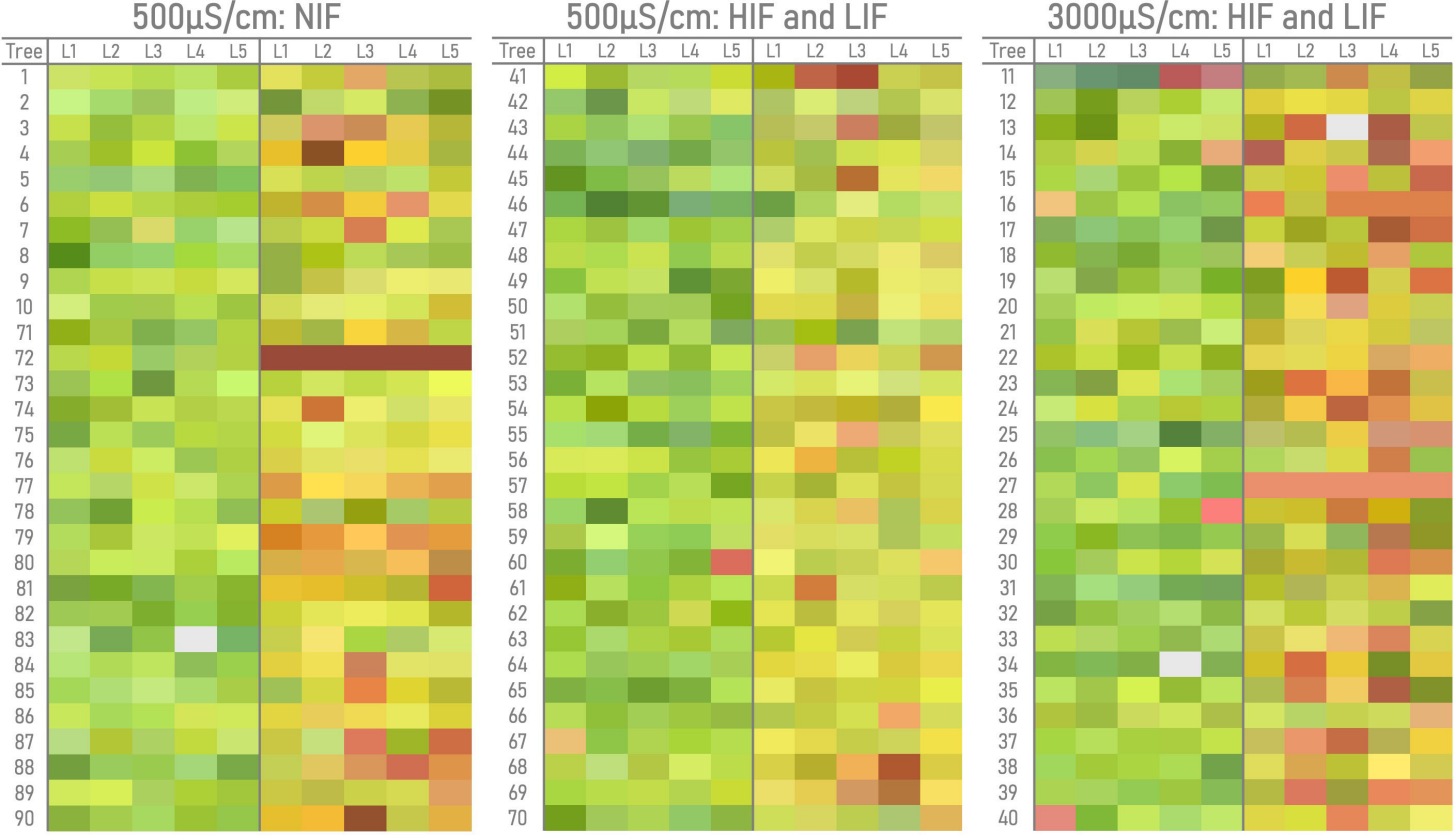
Colour change in five leaves (L1-L5) per tree from the start (left column) to the end (right column) of the experiment (five-month duration). Colour change was used as a visual tool to objectively assess deterioration. Fields with the colour grey indicate missing values.

Given the moderate sample size of the field data and the high site-to-site variance unraveled by the linear mixed-effects model, we set up a mesocosm experiment to examine predictors under more controlled circumstances. However, we noticed that H_2_O_2_ concentrations measured in the mesocosm were much lower than in the field (**Figure 4**). We also observed that all our trees showed signs of deterioration over the five months of exposure (**Figure 5**).

**Figure 6:**
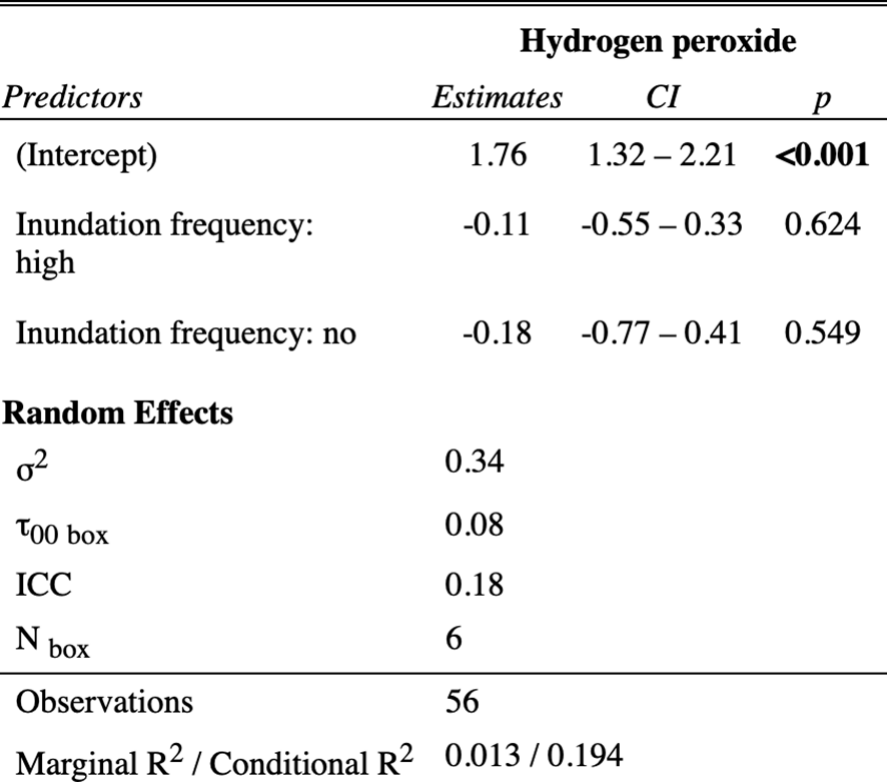
Results of the linear mixed-effects model for the mesocosm data comparing different inundation frequencies for oak trees exposed to fresh water. Hydrogen peroxide was measured in September 2024.

First, we evaluated whether inundation frequency (high, low, or no) by itself affects H_2_O_2_ concentrations (**Figure 6**). The model intercept (representing estimated log-transformed H_2_O_2_ concentrations at LIF) was 1.763 (SE = 0.222, *t* = 7.939). No significant effect of inundation frequency on H_2_O_2_ concentrations was detected but there was some variation between boxes (ICC = 0.18).

**Figure 7:**
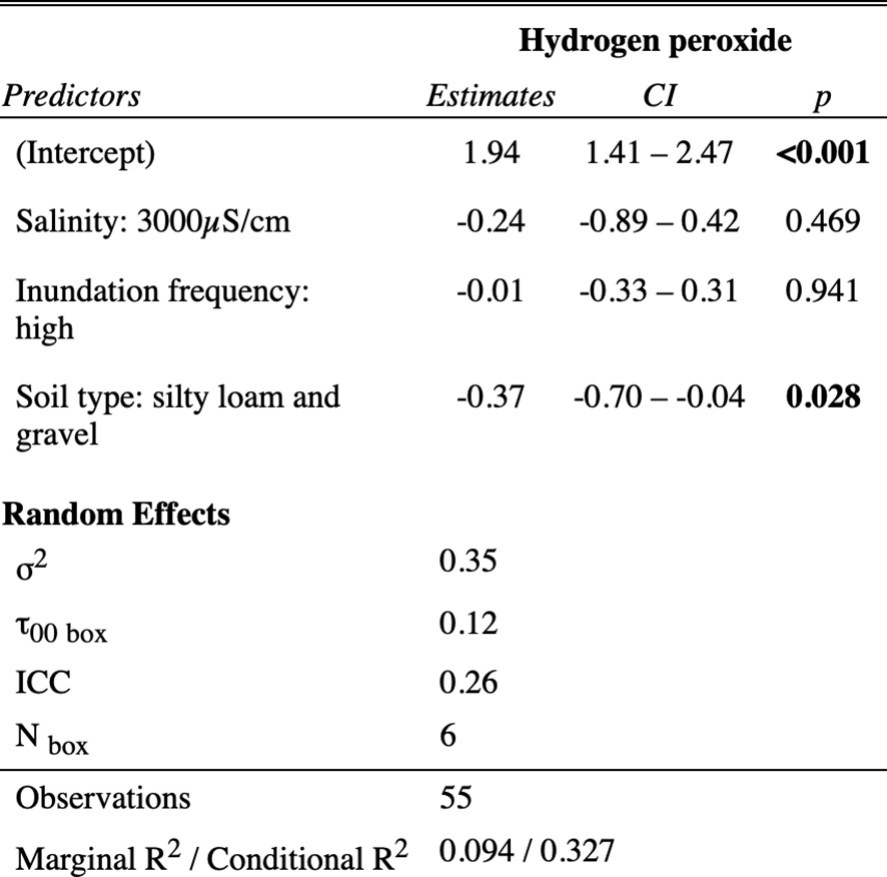
Results of the linear mixed-effects model for the mesocosm data comparing different inundation frequencies, salinities and soil types. Hydrogen peroxide was measured in September 2024.

In a second step we evaluated the impact of inundation frequency (low or high), salinity (500 or 3000µS/cm) and soil type (silty loam or silty loam mixed with gravel) on the H_2_O_2_ concentration (**Figure 7**). The model intercept was 1.940 (SE = 0.263, *t* = 7.386). While salinity had no effect on the H_2_O_2_ concentration, silty loam mixed with gravel did significantly decrease it (β = −0.372, SE = 0.164, *t* = −2.265, p = 0.028) and there was some variation between boxes (ICC = 0.26). The *post-hoc* simulation-based power analysis indicated moderate power to detect the effect of soil (62.1%, 95% CI: 59.0–65.1%). In contrast, power was very low for level (4.3%, 95% CI: 3.1–5.7%) and low for treatment (21.0%, 95% CI: 18.5–23.7%) suggesting that the study had limited sensitivity to detect the effects of level and treatment, and only moderate sensitivity for soil.

## 4 Discussion

### 4.1 Field campaign

Overall English oak deteriorated both in the field and the mesocosm setup, however, the underlying processes may not have been identical in both cases. The principal component analysis of the field data suggested that soil-related parameters (clay content, bulk density, soil conductivity), inundation frequency and elevation could be important predictors for oak tree survival. We therefore hypothesized that these may also underlie changes in leaf H_2_O_2_ concentrations, ultimately causing trees to die-off. However, H_2_O_2_ concentrations did not respond accordingly, making it difficult to deduce driving stressors from the field data with certainty.

Previous research on *Quercus macrocarpa* has identified H_2_O_2_ concentrations of up to 2370 ± 300 nmol/gFW under normal conditions (Cheeseman, 2006). None of our measurements exceeded this value (max. 1647 ± 418 nmol/gFW) suggesting limited H_2_O_2_ accumulation, which contrasts our observation of overall degradation and mortality. We suspect that stress and degradation can occur without a buildup of leaf H_2_O_2_, indicating that it may not adequately reflect the stress experienced by the trees in CRT areas. We also suspect that time of sampling plays an important role. Our sampling campaign took place end of summer while Cheeseman (2006) sampled in July, a period where higher photosynthetic activity is expected, inherently increasing H_2_O_2_ concentrations (Sharma et al., 2012). Future research should take this into consideration when using H_2_O_2_ as stress indicator.

Evaluation of the key predictors with respect to H_2_O_2_ concentrations only uncovered year as having a significant effect on H_2_O_2_ concentrations, with lower values measured in 2023 than in 2022. Our expectation was to see the reverse trend, since trees had been in the CRT areas for an additional year increasing the possibility of additional stress. Although not measured directly, we speculate that climatic factors could partially explain this trend. Hydrogen peroxide is produced during normal metabolic function (e.g. photosynthesis) as well as during stress conditions (e.g. drought, UV radiation, intense light) (Sharma et al., 2012; Das and Roychoudhury, 2014). With 2022 being the warmest year recorded in Belgium since the start of measurements in 1833 (Vlaamse-Milieumaatschappij, 2025), we wonder whether the heat contributed to the higher H_2_O_2_ concentrations measured in 2022 compared to 2023.

### 4.2 Mesocosm experiment

To circumvent the variability of the field, we decided to use a mesocosm setup to test distinct stressors under controlled conditions. The stressors were chosen based on the field data analysis as well as the real-life conditions found in the four CRT areas. To our surprise, H_2_O_2_ concentrations measured in the mesocosm experiment were even lower than the ones obtained in the field. For this we have two possible explanations. Number one, the conditions were not stressful enough to induce H_2_O_2_ accumulation even though all trees showed signs of deterioration over the five months of exposure (**Figure 4**). Or number two, young oak trees have a smaller physiological range resulting in overall lower H_2_O_2_ concentrations.

Our second explanation is backed by the findings of Singh and Stasolla (2016), who investigated H_2_O_2_ concentrations in three-month-old *Q. macrocarpa*. Although they investigated the same species as Cheeseman (2006), the H_2_O_2_ concentration measured in unstressed trees was 1.45 ± 0.45 nmol/gFW (Singh and Stasolla, 2016). This is more than a factor 1000 lower compared to the highest concentration measured by Cheeseman (2006) in more mature trees. Similarly, our H_2_O_2_ concentrations from the mesocosm experiment did not exceed 14.72 ± 3.35 nmol/gFW, several magnitudes lower than the concentrations found in the field. As such, tree age complicates the comparison of field and experimental data.

Despite the lower H_2_O_2_ concentrations measured in the mesocosm setup, we still expected the general trend of H_2_O_2_ concentrations to indicate possible stress. One of the specific objectives of this study was to investigate the effect of flooding on young oak trees, since flooding regimes are highly altered in CRT areas. Surprisingly, no difference in H_2_O_2_ concentrations were found between HIF, LIF and NIF after five months of intermitted flooding, within the constraints of our experimental design.

Results by Anderson and Pezeshki (1999) showed that intermitted flooding (5 days of flooding followed by 5 days of drainage) on six-month-old *Quercus nuttallii* Buckley and *Quercus michauxii* Nutt. did not reduce survival. One hundred percent of the *Q. nuttallii* and 96% of the *Q. michauxii* trees survived one month of intermitted flooding (Anderson and Pezeshki, 1999). Similarly, Angelov et al. (1996) observed that almost 90% of one year old *Q. michauxii* trees survived two years of intermitted flooding (dry period starting in June and flooding period starting in November). Interestingly this percentage was only 49% for *Quercus falcata* Michx (Angelov et al., 1996), revealing that different species of oak can have different sensitivities to flooding. Considering these findings and the absence of a clear H_2_O_2_ response to intermitted flooding in our study, it remains unclear whether English oak exhibits a degree of resistance to intermittent flooding, as described by Dreyer et al. (1991), or whether H_2_O_2_ is not a sufficiently sensitive proxy to capture this type of stress response.

Another objective of this study was to see how English oak would react to multiple stressor exposure. While no interaction effects between stressors (inundation frequency, salinity and soil type) were identified, a main effect of soil type was revealed. Trees planted in silty loam had higher H_2_O_2_ concentrations than the ones planted in silty loam mixed with gravel. However, non-significant results should be interpreted with caution as statistical power was relatively low for some predictors. The analysis therefore may lack sufficient sensitivity to detect small to moderate effects.

Overall, the presence of gravel can enhance drainage and decrease water logging of the soil. This could allow more oxygen to reach the tree roots, reducing oxidative stress and consequently H_2_O_2_ concentrations (Manghwar et al., 2024). Previous research on eleven month old English oak has demonstrated that waterlogging significantly increases root decay (Colinbelgrand et al., 1991) hinting at increased oxidative stress originating from water logging. Although this mechanism was not directly measured, we hypothesize that an improved drainage could be key to oak tree survival in CRT areas. However, drainage capacity is usually compromised in artificial CRT areas due to soil compaction from previous land use (Van Putte et al., 2020).

Contrary to our hypothesis, H_2_O_2_ concentrations in our mesocosm setup were not affected by salinity (3000µS/cm). Similar to flooding, this raises the question whether H_2_O_2_ is an appropriate proxy for this type of stress, as we did notice leaves changing color from green to brownish, often in the form of spots or brown edges, when exposed to higher salinity. This discoloration has been observed in other tree species exposed to excessive amounts of salt including *Acer platanoides* L., *Acer pseudoplatanus* L., *Fagus sylvatica* L., *Aesculus hippocastanum* L., and *Tilia cordata* Mill (Dmuchowski et al., 2022; Paludan-Muller et al., 2002).

According to Sehmer et al. (1995), some oak trees can store excessive salt (sodium and chloride ions) in old organs such as first flush leaves and first roots, allowing the second flush to develop normally. They observed leaf damage and necrotic leaf areas in English oak at a concentration of 40 mM (approx. 4000-4800 µS/cm) while Singh and Stasolla (2016) observed leaf injury in *Q. macrocarpa* from 25 mM NaCl (approx. 2500-3000 µS/cm) onwards. Given this information, we propose that the observed color change in leaves may indicate salt stress or a coping mechanism, despite the missing link with H_2_O_2_ concentrations.

### 4.3 Limitations

Several limitations should be considered when interpreting the results of this study. Firstly, the reliance on hydrogen peroxide (H_2_O_2_) as a single physiological indicator of stress represents an important constraint. Although H_2_O_2_ is widely recognized as a stress-related signaling molecule, our results showed no clear response to inundation frequency or salinity. Given the observed decline of trees in the field, this suggests that H_2_O_2_ alone may not adequately capture the physiological stress experienced by English oak in CRT areas, rather than indicating an absence of stress. This interpretation is supported by the high unexplained variance in our models, hinting at additional underlying mechanisms not captured. A broader selection of physiological markers including phytohormones (e.g. abscisic acid, indole-3-acetic acid, jasmonic acid, gibberellins), stress-related enzymes (e.g. ascorbate peroxidase, catalase, malondialdehyde) or other performance-related indicators (e.g. growth rate, survival, leaf area, number of leaves, leaf salt content) would likely provide a more comprehensive assessment of the stress response.

Secondly, the comparison between field observations and the mesocosm experiment proved challenging. Differences in tree age, size and physiology complicated direct comparisons, which is highlighted by the lack of consistent patterns between both systems. While the mesocosm was intended to explore potential mechanisms under controlled conditions, the results suggest that the complexity of the field conditions may limit the extent to which this is possible. Especially, the shorter exposure time in the mesocosm limited the amount of stress accumulation experienced in the field. Mature oaks in CRT areas have been exposed to changing conditions over multiple years, whereas the experimental period covered only a limited timeframe.

Finally, the number of replicates per treatment was constrained by logistical limitations inherent to working in CRT environments and maintaining mesocosm infrastructures. As a result, the statistical power to detect more subtle effects was limited, which may have contributed to the absence of significant effects in some of our models. Extending the duration of such experiments and increasing replication, where feasible, would help to better capture chronic stress responses and improve comparability across systems.

## 5 Conclusion

This study has identified that H_2_O_2_ concentrations measured in the field were affected by the time of sampling, while H_2_O_2_ concentrations measured in the mesocosm were affected by the soil type. Additionally, flooding and salinity did not seem to affect H_2_O_2_ concentrations individually or in combination with soil type. These results indicate that leaf H_2_O_2_, as measured in this study, did not reflect the deterioration of English oak observed in either the field or mesocosm setup. Moreover, observed discrepancies between field and mesocosm data suggest that key aspects of the stress response were not captured by the current setup relying solely on H_2_O_2_ as indicator and require further exploration.

This interpretation is further supported by the low proportion of variance (conditional R^2^) in H_2_O_2_ explained by the models. Rather than indicating the absence of stress, we suggest that these findings highlight the limited suitability of H_2_O_2_ as a standalone indicator of physiological stress in English oak under the conditions studied. We suggest that different tree life-history traits and the high complexity of dynamic CRT areas may contribute to this mismatch, although these factors were not directly tested in the present study.

We suggest that future research should not solely rely on H_2_O_2_ to assess the physiological stress response of English oak in CRT areas. Instead, we propose to include a broader range of physiological and performance-related indicators to provide a more comprehensive assessment and increase comparability across trees species as well as scientific studies. Such an approach would help to better capture the complexity of stress responses in such dynamic systems. We also propose to extend the period of evaluation for field and mesocosm experiments, if possible, to account for temporal variability and annual variation.

## Data Availability

The datasets presented in this article are not readily available and will be made available by the authors upon reasonable request.
